# A High Diet Quality Based on Dietary Recommendations Is Not Associated with Lower Incidence of Type 2 Diabetes in the Malmö Diet and Cancer Cohort

**DOI:** 10.3390/ijms17060901

**Published:** 2016-06-08

**Authors:** Emmanuel Mandalazi, Isabel Drake, Elisabet Wirfält, Marju Orho-Melander, Emily Sonestedt

**Affiliations:** Department of Clinical Sciences Malmö, Lund University, Clinical Research Centre, Jan Waldenströms gata 35, Malmö SE-20502, Sweden; emandalazi@yahoo.co.uk (E.M.); isabel.drake@med.lu.se (I.D.); elisabet.wirfalt@med.lu.se (E.W.); marju.orho-melander@med.lu.se (M.O.-M.)

**Keywords:** diet index, dietary pattern, type 2 diabetes, cohort

## Abstract

A high diet quality index based on Swedish nutrition recommendations has previously been associated with reduced risk of cardiovascular disease and mortality in the Malmö Diet and Cancer (MDC) cohort. The aim of the present study was to investigate whether this diet quality index was associated with the risk for type 2 diabetes. Of 26,868 participants (44–74 years) in the MDC cohort study, 3838 type 2 diabetes cases were identified from registers during 17 years of follow-up. A diet quality index (from a modified diet history method) was constructed based on adherence to the recommended intakes of saturated fat, polyunsaturated fat, fish, fiber, fruit and vegetables, and sucrose. After adjusting for potential confounders, we observed no significant association between the diet quality index and type 2 diabetes risk. The HR for the highest *vs.* lowest index category was 1.06 (95% CI: 0.94, 1.20; *p*-trend = 0.56). Because of the protective associations shown for cardiovascular disease and mortality, the specific dietary components that were chosen to represent adherence to the recommendations may be less applicable to type 2 diabetes risk.

## 1. Introduction

Type 2 diabetes prevalence is increasing worldwide [1]. Different approaches have been used to counter type 2 diabetes and have focused on lifestyles changes, including physical activity, moderate alcohol consumption, and zero tobacco usage [2]. Food habits have been shown to be related to the development and management of type 2 diabetes, for example by influencing glycemic control and insulin resistance [2,3,4,5,6,7]. Dietary patterns, rather than individual nutrients, such as prudent/healthy diets, the Mediterranean diet, Western diets, and traditional diets, have recently received a great deal of attention in assessing the association between diet and health. Because foods are consumed in combination and nutrients metabolized jointly, it might be more difficult to estimate the associations between individual foods or nutrients and disease incidence compared with the collective approach [6,8,9].

Against this background, a diet quality index based on Swedish nutrition recommendations and dietary guidelines was developed in the Malmö Diet and Cancer (MDC) study, which is a prospective population-based cohort in the south of Sweden [10]. A high diet quality index was found to be associated with a reduced risk of total mortality and cardiovascular disease (CVD) morbidity and mortality [8,11]. Although a healthy dietary pattern has been associated with reduced risk of type 2 diabetes elsewhere [3,5,7,12,13,14], the association between adherence to nutrition recommendations and type 2 diabetes risk has not been studied in the MDC cohort. We therefore aimed to investigate the association between diet quality, using the index and the risk of type 2 diabetes.

## 2. Results and Discussion

### 2.1. Description of Study Population

Table 1 summarizes the distribution of demographic, anthropometric and lifestyle baseline characteristics across the three categories of the diet quality index. A high diet quality index score was significantly associated with higher weight and BMI, being a non-smoker, having a high school or university education, having higher leisure-time physical activity, and being consumers of zero alcohol (both men and women). In addition, the diet quality index was inversely associated with fasting insulin in men. The diet quality index was significantly associated with all six included dietary components; a high diet quality was associated with higher intakes of dietary fiber, fruits and vegetables, fish and shellfish, and PUFAs and lower intakes of SFAs and sucrose (Table 1).

### 2.2. Diet Quality and Risk of Type 2 Diabetes

Across an average of 17 years (range: 0 to 24 years) of follow-up, 3838 individuals (1859 men and 1979 women) developed type 2 diabetes. We found no significant association between the diet quality index and the incidence of type 2 diabetes in the basic or multivariable model (Table 2). However, a positive association between the diet quality index and incidence of type 2 diabetes were observed in the multivariable model when not including BMI as a covariate (*p*-trend = 0.02). This association was stronger in women then in men; however, the interaction by sex was not statistically significant (*p* = 0.50). On the other hand, we observed an inverse association in the basic model after exclusion of those with potential unreliable dietary reports, *i.e.*, those reporting a substantial diet change in the past and energy misreporters (in total 36% of the study sample; *p*-trend = 0.18). However, this association was clearly attenuated after adjusting for potential confounders, including BMI (*p*-trend = 0.34). Only including diabetes cases identified in two or more registers did not have a major impact on the results. We also examined the relation with type 2 diabetes risk for the six individual dietary components separately. Those that adhered to the fiber recommendation had a lower risk of developing type 2 diabetes after adjusting for potential confounders and the other dietary components (Table 3). A low intake of SFAs (*i.e.*, <14 E%) was significantly associated with an increased risk of type 2 diabetes, but this association was clearly attenuated after exclusion of those reporting substantial diet changes and energy misreporters.

### 2.3. Discussion

The present study found no relationship between a diet quality index developed based on current nutrition recommendations and dietary guidelines in Sweden and the incidence of type 2 diabetes among non-diabetic participants of the MDC cohort across 17 years of follow-up. Because a high diet quality has previously been associated with decreased risk of CVD and mortality in the same cohort, the specific dietary components chosen to represent adherence to the recommendations may be better suited to predict CVD risk than type 2 diabetes risk. It might also be that recommendations overall rely more heavily on the association between diet and CVD than on the association between diet and type 2 diabetes.

Our study is the first to investigate adherence to Swedish nutrition recommendations and risk of type 2 diabetes. However, several previous cohort studies have investigated the role of overall diet quality in the risk of developing type 2 diabetes. A protective association between a “healthy” dietary patterns and type 2 diabetes has been reported in similar European cohorts [3,6,13,15]. Similar results have also been reported on diet quality scores among American men in which high scores were associated with a lower risk of type 2 diabetes [5]. The U.S.-based Nurses’ Health Study showed different results in reducing chronic disease risk depending on which score was used. For instance, adherence to the Healthy Eating Index (HEI-2005) was associated with a lower risk of type 2 diabetes, whereas the Alternative Healthy Eating Index (aHEI-2010) was found to be more strongly associated with both coronary heart disease and type 2 diabetes [16]. The protective effect was largely associated with dietary patterns that were characterized by high intakes of plant-based foods such as whole grains, moderate alcohol intake, and low intakes of red and processed meat, sodium, sugar-sweetened beverages, and *trans* fats.

We have previously seen that the recommended intakes of dietary fiber, fruit and vegetables, fish and shellfish, and sucrose were associated with a decreased risk of CVD [8]. The lack of association between adherence to the recommended intake of individual components of the diet quality index and incidence of type 2 diabetes was, however, not unexpected. A systematic review and a meta-analysis of five cohort studies did not find a significant protective association between fruit and vegetable intake and type 2 diabetes [17]. The tendency for increased risk among those who adhere to recommendations for SFA intake was not unexpected because a high intake of dairy products, one of the main source of SFAs in the MDC population, has been shown to reduce type 2 diabetes incidence [18,19]. In the MDC study, the high consumption of high-fat dairy products was shown to protect against type 2 diabetes [20]. In addition, recent evidence has shown that SFAs have a two-way effect on reducing and increasing the risk of type 2 diabetes because individual plasma phospholipid SFAs are heterogeneous; thus, it is imperative to understand their dietary intake sources and metabolic processes [21]. No association between fish and shellfish intake and the reduced risk of type 2 diabetes was found in a systematic review of 18 prospective cohorts [22]. However, other studies have reported a protective association between fish and shellfish intake and type 2 diabetes [23,24]. The long-chain omega-3 (*n*-3) fatty acids that are found in appreciable amounts in fish have been linked to potential protective effects against CVD and type 2 diabetes because they tend to counteract insulin resistance [24].

Our diet quality index may lack important dietary components associated with type 2 diabetes (e.g., protein, meat (especially processed meats), and sugar-sweetened beverages) and may, therefore, not fully account for the different food sources of fat, carbohydrates, and protein. For instance, we previously reported that high protein intake and different protein sources such as eggs, processed meat, and poultry increase the risk of type 2 diabetes in the MDC cohort [25]. We have also found an association between high intake of fiber, fiber-rich breads, and cereals and a decreased risk of type 2 diabetes. In addition, a high intake of sugar-sweetened beverages has consistently been associated with increased type 2 diabetes risk [26].

The present study has several notable strengths. The large sample size assured sufficient statistical power to study associations between dietary intake and the incidence of type 2 diabetes. The prospective nature, coupled with a dietary assessment method of high relative validity, was an important asset in minimizing bias. The MDC has unique dietary information; we combined a diet questionnaire with a seven-day food record and looked not only at the type of food being reported but also its preparation and cooking methods. The study was also able to control for many potential confounders. Furthermore, loss to follow-up in the MDC was very low, with a 99.3% follow-up rate. Our study also had limitations that need to be recognized. The one-time assessment of dietary intake and confounding factors at baseline are limitations because changes that occur during the follow-up period cannot be controlled. Although dietary intakes were collected carefully, measurement errors always occur that could have resulted in undetected associations [27]. In particular, dietary measurement errors are likely to lead to non-differential misclassification that may attenuate associations. This could be exacerbated by the under- or over-reporting of energy intake, though the total energy intake was considered in the analyses. Further, the possibility of excluding those who reported substantial dietary changes or were potential energy misreporters enabled us to examine the robustness of the data and the potential bias in estimates due to unstable food habits or misreporting errors. This was especially important because a high diet quality was associated with higher BMI as well as past food habit change. The potential bias was further illustrated by the significant positive association between the diet quality index and type 2 diabetes risk in the model not adjusting for BMI.

The study sample generally comprised older Swedish men and women, which could make it difficult to generalize the current findings to other age groups and other settings. Furthermore, very few participants adhered to the recommended levels of all six components [8,10]. The definition of high diet quality as adherence to five to six components is thus not fully reflective of adherence to the nutrition recommendations. In addition, dividing the individual components into adherence/non-adherence is crude and may affect the associations because the true variability of individual intakes may be hidden within the scores. Because the reported dietary intake was not likely to fully reflect the true intake, using specific cutoffs may result in a misclassification of individuals who are close to the cutoff limits [28]. Because each component of the diet quality index contributed an unweighted one point to the total score, it may be difficult to determine the resultant effect of each single component on health outcomes (*i.e.*, chronic diseases and mortality). Researchers have noted that although most diet quality indices are useful in evaluating diet quality, the ability to predict the risk of chronic disease may be moderate in some subjects because of an inappropriate selection of the components, selecting a small number of cutoff points for each component, or an equal contribution of all index items to the calculation of the total score [28,29]. Thus, the construction of the indices should include specific health or disease outcomes in relation to the dietary components. However, the diet quality index in the present study was developed to measure adherence to dietary recommendations [10]. Finally, residual confounding, which is a common problem in most observational studies, cannot be ruled out, despite controlling for potential confounders in this study.

This study found no association between a high-quality diet, as defined by a six-item diet quality index based on Swedish nutrition recommendations, and the risk of type 2 diabetes in the MDC cohort. Neither the overall diet quality index score nor its dietary components (except fiber) were significantly related to the risk of type 2 diabetes. Further studies need to examine the role of various individual dietary components (especially those that are not included in the index score) in the development of type 2 diabetes as well as overall dietary patterns that may help reduce the risk of this disease. In addition, because type 2 diabetes is to a high degree genetically determined, future studies should take genetic factors into account and examine the interactions between dietary habits and genetic susceptibility [30].

## 3. Materials and Methods

### 3.1. Study Population

The MDC study is a population-based prospective cohort of women born between 1923 and 1950 and men born between 1923 and 1945 who lived in Malmö and are proficient in the Swedish language. Baseline examinations were performed between 1991 and 1996 [31]. The identification of potential study participants for the MDC cohort was done through the Swedish national population registries. Out of 74,138 men and women eligible for the study, 28,098 individuals completed the baseline examinations including dietary collection. Further details on the recruitment process are presented elsewhere [31,32]. A random sample of those participating between October 1991 and February 1994 were invited to provide fasting blood samples for additional measurements including blood glucose and plasma insulin [33]. For the present study, we excluded all subjects with diabetes at baseline (identified from registers (see below) or self-reported diabetes diagnosis or medication), resulting in a total study population of 26,868 individuals (62% females). The Ethical Committee at Lund University approved the study (Lund University 51–90, Lund, Sweden), and informed written consent was obtained from each participant during recruitment.

### 3.2. Type 2 Diabetes Case Ascertainment

Type 2 diabetes cases were identified from seven registers as well as information from MDC (baseline and rescreening) and the Malmö Preventive Project [34] until the end of the follow-up period (31 December 2014). The National Diabetes Register [35] and the regional Diabetes 2000 Register [36] required a proven diagnosis by a physician at the hospital based on international diagnosis standards (fasting plasma glucose concentration ≥7.0 mmol/L, measured twice) [37]. For cases that were not diagnosed at a hospital, the local HbA1c register from the Department of Clinical Chemistry, Skåne University Hospital, Malmö, Sweden, was used, which has analyzed all HbA1c samples obtained from institutional and non-institutional care in Malmö since 1988. Individuals with at least two HbA1c values greater than 6.0% based on the Swedish Mono-S standardization system (corresponding to 6.9% with the U.S. National Glycohemoglobin Standardization Program and 52 mmol/mol with the International Federation of Clinical Chemistry and Laboratory Medicine (IFCC) units) [38,39] were categorized as diabetes cases. We also used other sources to identify diabetes cases. In the National Patient Register and the Swedish Cause of Death Register, the International Classification of Diseases (ICD) 10 codes E10–E14 and O244–O249 were used to identify diabetes patients. In the Prescribed Drug Register, the Anatomic Therapeutic Chemical classification (ATC) code A10 was used to identify diabetes patients. Information from the Malmö Preventive Project cohort (baseline screening 1997–1992, six-year rescreening 1981–1989, and rescreening 2002–2006) [34] and the MDC cohort (baseline screening 1991–1996, baseline screening cardiovascular cohort 1992–1994, five-year rescreening 1997–2001, and cardiovascular rescreening 2007–2012) [40] were also used to identify diabetes cases. Individuals with fasting blood glucose ≥6.5 mmol/L or fasting plasma glucose ≥7.0 mmol/L (verified with plasma glucose or oral glucose tolerance test (OGTT) in subsequent examination), ≥11 mmol/L 2-h after OGTT, intake of diabetes medication (A10 drugs), or who have reported having diabetes in a questionnaire were identified as incident diabetes cases. We excluded cases with incident type 1 diabetes (*n* = 144), Latent Autoimmune Diabetes in Adults (LADA) (*n* = 10), secondary diabetes (*n* = 1), and other diabetes conditions (*n* = 9).

### 3.3. Dietary Assessment

Dietary information was collected at baseline using a modified diet history methodology that included a combination of (a) a seven-day diary for lunch and dinner meals and cold beverages; (b) a 168-food item questionnaire with frequency and portion sizes of other foods during the previous year; and (c) a 1-h interview on portion sizes, cooking practices, and recipes for foods recorded in the seven-day diary [41]. The food intake (g/day) was calculated based on frequency and portion size estimates from the questionnaire and food diary (and interview). Energy and nutrient intakes were computed based on the PC Kost2-93 of the National Food Administration in Uppsala, Sweden. The validity and reproducibility of the data have been published and showed the relatively high validity of the MDC method compared with other dietary assessment methods used in similar populations [42,43,44].

A diet quality index based on the 2005 Swedish Nutrition Recommendations and the Swedish Dietary Guidelines was developed and has been tested within the MDC cohort [10]. The index consists of six components: energy percentage (E%) from saturated fat (SFA), E% from polyunsaturated fat (PUFA), intake of fish and shellfish (g/week), intake of dietary fiber (g/MJ), intake of fruit and vegetables (g/day), and E% from sucrose. The E% calculations were based on the non-alcohol energy intake because alcohol could change the diet and influence the health outcomes. The recommended intake levels were set at specific cutoff points for each of the diet quality index components as follows: SFA ≤ 14 E%, PUFA 5–10 E%, fish and shellfish ≥300 g/week, dietary fiber 2.4–3.6 g/MJ, fruit and vegetables ≥400/day, and sucrose ≤10 E%. Because only 2% of the cohort had an intake below the recommended intake for SFA (10 E%), the cutoff was adjusted to 1 standard deviation (SD) above the mean intake (14 E%). The recommended intake for dietary fiber is 25–35 g/day (approximately 3 g/MJ), and using a similar approach resulted in a range of dietary fiber (±1 SD of the population mean) from 2.4 to 3.6 g/MJ. There was no change in recommendations for the included components in the updated 2012 Nordic Nutrition Recommendations [9]. One point was assigned for individuals who reached the predefined cutoff limit for each component and zero points for those that had intake outside the recommended cutoff value. This led to the construction of a total diet quality index score that ranged from 0 to 6, which was categorized into three groups: low (0–1 points), medium (2–4 points) and high (5–6 points) [10,11].

### 3.4. Assessment of Other Covariates

Gender and age data were obtained from the Swedish national tax and civil registration system. A self-administered questionnaire was used to compile lifestyle and socioeconomic information for all study participants at baseline [45]. Smoking status was recorded as never, former, or current smokers (including irregular smokers). Leisure-time physical activity was determined using a questionnaire adapted from the Minnesota Leisure-Time Physical Activity Questionnaire [46]. Subjects were asked to report the average number of minutes per week spent on each activity during each of the four seasons. A final physical activity score was obtained by multiplying the number of minutes for each activity with an activity-specific factor, and divided into quartiles. Educational level was defined by the duration of completed education attained, *i.e.*, elementary, primary and secondary, upper secondary, further education without a degree, and university degree. Alcohol consumption was recorded into six categories. Participants who did not report any alcohol intake in the seven-day food diary and who indicated no alcohol intake over the previous year in the questionnaire were classified as zero-consumers. Otherwise, gender-specific quintiles were constructed based on the alcohol intake reported in the seven-day diary, with cutoff levels of 3.3, 9.1, 15.7, and 25.7 g/day for men and 0.9, 4.3, 8.1, and 14.0 g/day for women. Weight (kg), height (cm), and waist measurements (cm) were taken and body fat percentage was measured using bioelectric impedance method (single-frequency analyzer, bioelectric impedance analysis (BIA) 103; JRL Systems, Detroit, MI, USA). BMI was computed as weight (kg) divided by height squared (m^2^).

Energy misreporting among study participants was defined as those individuals with a ratio of reported energy intake to estimated basal metabolic rate outside the 95% confidence interval (CI) of the calculated physical activity level (total energy expenditure estimated from information on physical activity at work, during leisure time, household work, estimated sleeping hours, self-care, and passive time divided by basal metabolic rate) [47]. Participants reporting a substantial change in their eating habits in the past due to illness or other reasons were identified from the questionnaire [48]. The season of baseline data collection (January–March, April–June, July–September, October–December) was also created as a categorical variable. The dietary variables were also adjusted for a variable called ‘method version’ to account for a new coding system of dietary data introduced in September 1994 that helped reduce the interview time (from 60 to 45 min) [44]. This resulted in two slightly different method versions (before or after September 1994), but the ranking of individuals was not affected by this change.

### 3.5. Statistical Analysis

The SPSS statistical software packages (version 22; IBM Corporation, Armonk, NY, USA) and Stata/SE (version 12.2, StatCorp LP, College Station, TX, USA) were used for all of the analyses. All tests were two-sided, and *p*-values <0.05 were considered statistically significant. The χ-square tests (for categorical variables) and ANOVA (for continuous variables) were used to assess differences in baseline characteristics by diet quality index. Cox proportional hazards regression analysis was used to estimate hazard ratios (HR) and 95% CI for type 2 diabetes incidence by total diet quality scores and by adherence *versus* non-adherence to the individual diet quality index components. The proportional hazards assumption was assessed using Schoenfeld test. To fulfill the proportional hazards assumption, the model was stratified for age and BMI quartiles. Person time was calculated from baseline up to diagnosis of type 2 diabetes, emigration, loss to follow-up, or end of follow-up on 31 December 2014. The multivariable adjusted models included the following potential confounders: age (stratified by quartiles), sex, total energy intake, diet assessment method version, season when interviews were conducted, educational level, leisure-time physical activity, smoking status, alcohol consumption, and BMI (stratified by quartiles). The potential confounders were identified based on a literature search [6,26,49]. We also ran the analyses without BMI in the model. In sensitivity analyses we excluded those who were classified as energy misreporters and those who reported substantial change in food habits in the past (*n* = 9637) [47,48]. Trends across diet index categories were tested with diet quality index as continuous variable (0 to 6). We also ran the analyses including only type 2 diabetes cases identified from more than one source (*n* = 3015 cases).

## Figures and Tables

**Table 1:** Baseline characteristics of men (*n* = 10,413) and women (*n* = 16,455) in the Malmö Diet and Cancer cohort (1991–1996) by categories of diet quality index score.

Variables	Men				Women			
	Low (0–1)	Medium (2–4)	High (5–6)	*p*-Value	Low (0–1)	Medium (2–4)	High (5–6)	*p*-Value
Number of Participants	1536	7756	1121		2661	11,450	2344	
Age (years)	59.1 (7.3)	59.0 (7.0)	59.6 (6.8)	0.05	57.2 (8.2)	57.2 (7.9)	57.7 (7.4)	0.02
Height (cm)	176.0 (6.6)	176.5 (6.6)	176.4 (6.7)	0.03	163.4 (6.1)	163.8 (6.0)	163.6 (6.1)	0.02
Weight (kg)	80.0 (12.0)	81.7 (12.0)	82.0 (11.8)	<0.001	66.6 (11.6)	67.9 (11.5)	68.5 (11.5)	<0.001
Body mass index (kg/m^2^)	25.8 (3.4)	26.2 (3.4)	26.3 (3.3)	<0.001	24.9 (4.1)	25.3 (4.2)	25.6 (4.1)	<0.001
Waist circumference (cm)	92.8 (9.9)	93.6 (9.9)	93.3 (9.7)	0.03	77.0 (10.2)	77.6 (10.3)	77.7 (10.1)	0.01
Body fat (%)	20.7 (5.0)	20.7 (5.0)	20.5 (4.7)	0.63	30.4 (5.1)	30.7 (4.9)	30.9 (4.9)	0.01
Plasma glucose (mmol/L)	5.84 (0.81)	5.83 (0.94)	5.77 (0.98)	0.61	5.53 (0.61)	5.51 (0.72)	5.48 (0.68)	0.50
Plasma insulin (pmol/L)	56.9 (57.2)	51.6 (59.7)	47.8 (36.2)	0.02	43.8 (26.7)	42.4 (37.0)	43.4 (41.8)	0.21
Energy intake (MJ)	11.1 (2.9)	11.1 (2.9)	10.8 (2.6)	<0.001	8.7 (2.1)	8.6 (2.1)	8.3 (1.9)	<0.001
Saturated fat (E%)	18.3 (3.7)	16.5 (3.8)	12.5 (2.4)	<0.001	18.1 (3.5)	16.3 (3.6)	13.0 (2.4)	<0.001
Polyunsaturated fat (E%)	5.2 (1.7)	6.3 (1.5)	6.3 (1.3)	<0.001	5.0 (1.5)	5.9 (1.5)	6.2 (1.3)	<0.001
Sucrose (E%)	11.3 (4.3)	7.8 (3.4)	7.0 (2.2)	<0.001	11.3 (3.9)	8.5 (3.2)	7.6 (2.0)	<0.001
Fiber (g/MJ)	1.65 (0.36)	2.00 (0.53)	2.86 (0.54)	<0.001	1.80 (0.36)	2.29 (0.59)	3.02 (0.57)	<0.001
Fish and shellfish (g/week)	175 (157)	361 (276)	539 (329)	<0.001	173 (129)	297 (209)	451 (223)	<0.001
Fruit and vegetables (g/day)	241 (107)	335 (173)	548 (183)	<0.001	272 (106)	388 (175)	568 (168)	<0.001
Level of energy reporting				0.04				<0.001
Under-reporters	169 (11.0)	906 (11.7)	151 (13.5)		358 (13.5)	1984 (17.3)	517 (22.1)	
Adequate reporters	1302 (84.8)	6564 (84.6)	943 (84.1)		2198 (82.6)	9136 (79.8)	1797 (76.7)	
Over-reporters	65 (4.2)	289 (3.7)	27 (2.4)		105 (3.9)	330 (2.9)	30 (1.3)	
Substantial diet change in the past	229 (15.0)	1456 (18.8)	424 (37.8)	<0.001	437 (16.4)	2524 (22.1)	917 (39.2)	<0.001
Season of diet collection				0.11				0.13
January–March	390 (25.4)	1756 (22.6)	245 (21.9)		638 (24.0)	2506 (21.9)	542 (23.1)	
April–June	441 (28.7)	2386 (30.8)	362 (32.3)		737 (27.7)	3370 (29.4)	702 (29.9)	
July–September	206 (13.4)	1109 (14.3)	142 (12.7)		397 (14.9)	1745 (15.2)	326 (13.9)	
October–December	499 (32.5)	2505 (32.3)	372 (33.2)		889 (33.4)	3829 (33.4)	774 (33.0)	
Educational level				<0.001				<0.001
Elementary	811 (53.1)	3509 (45.3)	443 (39.6)		1167 (43.9)	4403 (38.6)	826 (35.3)	
Primary and secondary	284 (18.6)	1513 (19.5)	243 (21.7)		775 (29.2)	3491 (30.6)	730 (31.2)	
Upper secondary	142 (9.3)	955 (12.3)	142 (12.7)		193 (7.3)	803 (7.0)	156 (6.7)	
Further education without a degree	117 (7.7)	721 (9.3)	122 (10.9)		206 (7.8)	948 (8.3)	240 (10.3)	
University	174 (11.4)	1043 (13.5)	169 (15.1)		317 (11.9)	1771 (15.5)	289 (16.6)	
Alcohol consumption				<0.001				<0.001
Zero consumers	101 (6.6)	303 (3.9)	50 (4.5)		283 (10.6)	781 (6.8)	142 (6.1)	
Quintile 1	387 (25.2)	1364 (17.6)	213 (19.0)		615 (23.1)	1998 (17.4)	386 (16.5)	
Quintile 2	294 (19.1)	1442 (18.6)	231 (20.6)		511 (19.2)	2059 (18.0)	456 (19.5)	
Quintile 3	262 (17.1)	1507 (19.4)	244 (21.8)		438 (16.5)	2163 (18.9)	460 (19.6)	
Quintile 4	271 (17.6)	1529 (19.7)	216 (19.3)		399 (15.0)	2212 (19.3)	467 (19.9)	
Quintile 5	221 (14.4)	1611 (20.8)	167 (14.9)		415 (15.6)	2237 (19.5)	433 (18.5)	
Smoking status				<0.001				<0.001
Current	596 (38.8)	2245 (28.9)	164 (14.6)		998 (37.5)	3219 (28.1)	413 (17.6)	
Former	552 (38.8)	3339 (28.9)	554 (14.6)		614 (23.1)	3148 (27.5)	793 (33.8)	
Never	387 (35.9)	2168 (43.1)	403 (49.4)		1047 (39.3)	5081 (44.4)	1137 (48.5)	
Leisure-time physical activity				<0.001				<0.001
Quartile 1	509 (33.5)	1954 (25.4)	170 (15.2)		821 (31.0)	2815 (24.8)	404 (17.3)	
Quartile 2	366 (24.1)	1887 (24.5)	242 (21.7)		677 (25.6)	2923 (25.7)	578 (24.8)	
Quartile 3	303 (19.9)	1843 (23.9)	310 (27.8)		612 (23.1)	2942 (25.9)	660 (28.3)	
Quartile 4	343 (22.6)	2021 (26.2)	395 (35.4)		536 (20.3)	2688 (23.6)	693 (29.7)	

Data are presented as mean (SD) or *n* (%). Glucose concentrations were available for 2060 males and 3027 females; insulin concentrations were available for 1988 males and 2923 females. *p*-Value was for ln-transformed plasma insulin concentrations.

**Table 2:** Hazard ratio (HR) and 95% confidence interval (CI) for incident type 2 diabetes by categories of diet quality index score among men (*n* = 10,413) and women (*n* = 16,455) in the Malmö Diet and Cancer cohort (1991–2014).

Model	Low (0–1)	Medium (2–4)	High (5–6)	*p*-Trend
All				
Cases/person-years	563/70,894	2763/332,587	512/62,071	
Basic model ^a^	1.00	1.01 (0.92–1.11)	1.01 (0.90–1.14)	0.60
Multivariable model excl. BMI ^b^	1.00	1.10 (1.00–1.20)	1.17 (1.03–1.32)	0.02
Multivariable model ^c^	1.00	1.03 (0.94–1.13)	1.06 (0.94–1.20)	0.56
Adequate reporters in basic model ^d^	1.00	0.99 (0.87–1.11)	0.91 (0.77–1.08)	0.03
Adequate reporters in multivariable model excl. BMI ^e^	1.00	1.10 (0.98–1.23)	1.09 (0.91–1.29)	0.70
Adequate reporters in multivariable model ^f^	1.00	1.02 (0.91–1.15)	1.01 (0.85–1.20)	0.34
Men				
Cases/person-years	255/25,165	1399/126,887	205/19,136	
Basic model ^a^	1.00	1.04 (0.91–1.18)	0.99 (0.82–1.19)	0.55
Multivariable model excl. BMI ^b^	1.00	1.10 (0.96–1.26)	1.10 (0.91–1.32)	0.39
Multivariable model ^c^	1.00	1.04 (0.91–1.19)	1.02 (0.84–1.23)	0.96
Adequate reporters in basic model ^d^	1.00	1.05 (0.89–1.24)	0.97 (0.76–1.25)	0.40
Adequate reporters in multivariable model excl. BMI ^e^	1.00	1.14 (0.97–1.35)	1.15 (0.89–1.48)	0.98
Adequate reporters in multivariable model ^f^	1.00	1.06 (0.89–1.25)	1.06 (0.82–1.37)	0.89
Women				
Cases/person-years	308/46,729	1364/205,700	307/42,936	
Basic model ^a^	1.00	0.99 (0.87–1.12)	1.03 (0.88–1.21)	0.88
Multivariable model excl. BMI ^b^	1.00	1.09 (0.97–1.24)	1.23 (1.04–1.44)	0.02
Multivariable model ^c^	1.00	1.02 (0.90–1.16)	1.10 (0.93–1.29)	0.40
Adequate reporters in basic model ^d^	1.00	0.95 (0.81–1.11)	0.86 (0.68–1.08)	0.02
Adequate reporters in multivariable model excl. BMI ^e^	1.00	1.06 (0.90–1.24)	1.05 (0.83–1.33)	0.89
Adequate reporters in multivariable model ^f^	1.00	0.99 (0.85–1.17)	0.97 (0.77–1.23)	0.26

^a^ Adjustments for age (stratified), sex, total energy intake, diet assessment method, and season when interviews were conducted; ^b^ Adjustments for age (stratified), sex, total energy intake, diet assessment method version, season when interviews were conducted, educational level, leisure-time physical activity, smoking status, and alcohol consumption; ^c^ Adjustments for age (stratified), sex, total energy intake, diet assessment method version, season when interviews were conducted, educational level, leisure-time physical activity, smoking status, alcohol consumption, and BMI (stratified); ^d^ Adjustments as in basic model and excluding both diet changers and energy misreporters (*n* = 9637) from the analyses; ^e^ Adjustments as in multivariable model excl. BMI and excluding both diet changers and energy misreporters (*n* = 9637) from the analyses; ^f^ Adjustments as in multivariable model and excluding both diet changers and energy misreporters (*n* = 9637) from the analyses.

**Table 3:** Hazard ratio (HR) and 95% confidence interval (CI) for incident type 2 diabetes by adherence to diet quality index components among participants in the Malmö Diet and Cancer cohort (1991–2014).

Dietary Components	Non-Adherence	Adherence
Saturated fat	>14 E%	≤14 E%
Mutually adjusted multivariable model excl. BMI ^a^	1.00	1.19 (1.10–1.29)
Mutually adjusted multivariable model ^b^	1.00	1.14 (1.06–1.24)
Adequate reporters in mutually adjusted multivariable model ^c^	1.00	1.02 (0.92–1.14)
Polyunsaturated fat	<5 E% or >10 E%	5–10 E%
Mutually adjusted multivariable model excl. BMI ^a^	1.00	1.04 (0.97–1.12)
Mutually adjusted multivariable model ^b^	1.00	1.02 (0.95–1.09)
Adequate reporters in mutually adjusted multivariable model ^c^	1.00	1.05 (0.95–1.15)
Sucrose	≥10 E%	≤10 E%
Mutually adjusted multivariable model excl. BMI ^a^	1.00	1.07 (1.00–1.16)
Mutually adjusted multivariable model ^b^	1.00	1.01 (0.94–1.09)
Adequate reporters in mutually adjusted multivariable model ^c^	1.00	1.02 (0.92–1.12)
Fiber	≤2.4 or >3.6 g/MJ	2.4–3.6 g/MJ
Mutually adjusted multivariable mode excl. BMI ^a^	1.00	0.85 (0.78–0.92)
Mutually adjusted multivariable model ^b^	1.00	0.91 (0.84–0.99)
Adequate reporters in mutually adjusted multivariable model ^c^	1.00	0.89 (0.79–1.00)
Fish and shellfish	≤300 g/week	≥300 g/week
Mutually adjusted multivariable mode excl. BMI ^a^	1.00	1.02 (0.95–1.09)
Mutually adjusted multivariable model ^b^	1.00	1.01 (0.94–1.08)
Adequate reporters in mutually adjusted multivariable model ^c^	1.00	0.99 (0.90–1.08)
Fruits and vegetables	≤400 g/day	≥400 g/day
Mutually adjusted multivariable mode excl. BMI ^a^	1.00	0.98 (0.91–1.05)
Mutually adjusted multivariable model ^b^	1.00	0.99 (0.92–1.07)
Adequate reporters in mutually adjusted multivariable model ^c^	1.00	1.01 (0.91–1.11)

^a^ Adjusted for age (stratified), sex, total energy intake, diet assessment method version, season when interviews were conducted, educational level, leisure-time physical activity, smoking status, alcohol consumption and mutual adjustment for the other diet quality index components; ^b^ Adjusted for age (stratified), sex, total energy intake, diet assessment method version, season when interviews were conducted, educational level, leisure-time physical activity, smoking status, alcohol consumption, and BMI (stratified) and mutual adjustment for the other diet quality index components; ^c^ Adjusted for the same variables as in the mutually adjusted multivariable model but excluding both diet changers and energy misreporters (*n* = 9637) from the analyses.

## References

[1] International Diabetes Federation (2015). Diabetes Atlas.

[2] Lazarou C., Panagiotakos D., Matalas A.L. (2012). The role of diet in prevention and management of type 2 diabetes: Implications for public health. Crit. Rev. Food Sci. Nutr..

[3] Heidemann C., Hoffmann K., Spranger J., Klipstein-Grobusch K., Mohlig M., Pfeiffer A.F., Boeing H. (2005). A dietary pattern protective against type 2 diabetes in the European prospective investigation into cancer and nutrition (EPIC)—Potsdam study cohort. Diabetologia.

[4] Shirani F., Salehi-Abargouei A., Azadbakht L. (2013). Effects of dietary approaches to stop hypertension (DASH) diet on some risk for developing type 2 diabetes: A systematic review and meta-analysis on controlled clinical trials. Nutrition.

[5] De Koning L., Chiuve S.E., Fung T.T., Willett W.C., Rimm E.B., Hu F.B. (2011). Diet-quality scores and the risk of type 2 diabetes in men. Diabetes Care.

[6] Montonen J., Knekt P., Harkanen T., Jarvinen R., Heliovaara M., Aromaa A., Reunanen A. (2005). Dietary patterns and the incidence of type 2 diabetes. Am. J. Epidemiol..

[7] Schwingshackl L., Hoffmann G. (2015). Diet quality as assessed by the healthy eating index, the alternate healthy eating index, the dietary approaches to stop hypertension score, and health outcomes: A systematic review and meta-analysis of cohort studies. J. Acad. Nutr. Diet..

[8] Hlebowicz J., Drake I., Gullberg B., Sonestedt E., Wallstrom P., Persson M., Nilsson J., Hedblad B., Wirfalt E. (2013). A high diet quality is associated with lower incidence of cardiovascular events in the malmo diet and cancer cohort. PLoS ONE.

[9] Nordic Council of Ministers (2014). Nordic Nutrition Recemmendations 2012—Intergrating Nutrition and Physical Activity.

[10] Drake I., Gullberg B., Ericson U., Sonestedt E., Nilsson J., Wallstrom P., Hedblad B., Wirfalt E. (2011). Development of a diet quality index assessing adherence to the swedish nutrition recommendations and dietary guidelines in the malmo diet and cancer cohort. Public Health Nutr..

[11] Drake I., Gullberg B., Sonestedt E., Wallstrom P., Persson M., Hlebowicz J., Nilsson J., Hedblad B., Wirfalt E. (2013). Scoring models of a diet quality index and the predictive capability of mortality in a population-based cohort of swedish men and women. Public Health Nutr..

[12] Maghsoudi Z., Azadbakht L. (2012). How dietary patterns could have a role in prevention, progression, or management of diabetes mellitus? Review on the current evidence. J. Res. Med. Sci..

[13] InterAct Consortium (2014). Adherence to predefined dietary patterns and incident type 2 diabetes in European populations: EPIC-interAct study. Diabetologia.

[14] Lacoppidan S., Kyro C., Loft S., Helnaes A., Christensen J., Hansen C.P., Dahm C.C., Overvad K., Tjonneland A., Olsen A. (2015). Adherence to a healthy nordic food index is associated with a lower risk of type-2 diabetes-the danish diet, cancer and health cohort study. Nutrients.

[15] InterAct Consortium (2011). Mediterranean diet and type 2 diabetes risk in the European prospective investigation into cancer and nutrition (EPIC) study: The interact project. Diabetes Care.

[16] Chiuve S.E., Fung T.T., Rimm E.B., Hu F.B., McCullough M.L., Wang M., Stampfer M.J., Willett W.C. (2012). Alternative dietary indices both strongly predict risk of chronic disease. J. Nutr..

[17] Hamer M., Chida Y. (2007). Intake of fruit, vegetables, and antioxidants and risk of type 2 diabetes: Systematic review and meta-analysis. J. Hypertens..

[18] Tong X., Dong J.Y., Wu Z.W., Li W., Qin L.Q. (2011). Dairy consumption and risk of type 2 diabetes mellitus: A meta-analysis of cohort studies. Eur. J. Clin. Nutr..

[19] Margolis K.L., Wei F., de Boer I.H., Howard B.V., Liu S., Manson J.E., Mossavar-Rahmani Y., Phillips L.S., Shikany J.M., Tinker L.F. (2011). A diet high in low-fat dairy products lowers diabetes risk in postmenopausal women. J. Nutr..

[20] Ericson U., Hellstrand S., Brunkwall L., Schulz C.A., Sonestedt E., Wallstrom P., Gullberg B., Wirfalt E., Orho-Melander M. (2015). Food sources of fat may clarify the inconsistent role of dietary fat intake for incidence of type 2 diabetes. Am. J. Clin. Nutr..

[21] Forouhi N.G., Koulman A., Sharp S.J., Imamura F., Kroger J., Schulze M.B., Crowe F.L., Huerta J.M., Guevara M., Beulens J.W. (2014). Differences in the prospective association between individual plasma phospholipid saturated fatty acids and incident type 2 diabetes: The EPIC-interAct case-cohort study. Lancet Diabetes Endocrinol..

[22] Wu J.H., Micha R., Imamura F., Pan A., Biggs M.L., Ajaz O., Djousse L., Hu F.B., Mozaffarian D. (2012). Omega-3 fatty acids and incident type 2 diabetes: A systematic review and meta-analysis. Br. J. Nutr..

[23] Nanri A., Mizoue T., Noda M., Takahashi Y., Matsushita Y., Poudel-Tandukar K., Kato M., Oba S., Inoue M., Tsugane S. (2011). Fish intake and type 2 diabetes in japanese men and women: The Japan public health center-based prospective study. Am. J. Clin. Nutr..

[24] Zheng J.S., Huang T., Yang J., Fu Y.Q., Li D. (2012). Marine *n*-3 polyunsaturated fatty acids are inversely associated with risk of type 2 diabetes in Asians: A systematic review and meta-analysis. PLoS ONE.

[25] Ericson U., Sonestedt E., Gullberg B., Hellstrand S., Hindy G., Wirfalt E., Orho-Melander M. (2013). High intakes of protein and processed meat associate with increased incidence of type 2 diabetes. Br. J. Nutr..

[26] InterAct Consortium (2013). Consumption of sweet beverages and type 2 diabetes incidence in European adults: Results from EPIC-interAct. Diabetologia.

[27] Sempos C.T., Liu K., Ernst N.D. (1999). Food and nutrient exposures: What to consider when evaluating epidemiologic evidence. Am. J. Clin. Nutr..

[28] Waijers P.M., Feskens E.J., Ocke M.C. (2007). A critical review of predefined diet quality scores. Br. J. Nutr..

[29] Kourlaba G., Panagiotakos D.B. (2009). Dietary quality indices and human health: A review. Maturitas.

[30] Franks P.W., Pearson E., Florez J.C. (2013). Gene-environment and gene-treatment interactions in type 2 diabetes: Progress, pitfalls, and prospects. Diabetes Care.

[31] Manjer J., Carlsson S., Elmstahl S., Gullberg B., Janzon L., Lindstrom M., Mattisson I., Berglund G. (2001). The malmo diet and cancer study: Representativity, cancer incidence and mortality in participants and non-participants. Eur. J. Cancer Prev..

[32] Berglund G., Elmstahl S., Janzon L., Larsson S.A. (1993). The malmo diet and cancer study. Design and feasibility. J. Intern. Med..

[33] Hedblad B., Nilsson P., Engstrom G., Berglund G., Janzon L. (2002). Insulin resistance in non-diabetic subjects is associated with increased incidence of myocardial infarction and death. Diabet. Med..

[34] Berglund G., Nilsson P., Eriksson K.F., Nilsson J.A., Hedblad B., Kristenson H., Lindgarde F. (2000). Long-term outcome of the malmo preventive project: Mortality and cardiovascular morbidity. J. Intern. Med..

[35] Cederholm J., Eeg-Olofsson K., Eliasson B., Zethelius B., Nilsson P.M., Gudbjornsdottir S. (2008). Risk prediction of cardiovascular disease in type 2 diabetes: A risk equation from the swedish national diabetes register. Diabetes Care.

[36] Lindholm E., Agardh E., Tuomi T., Groop L., Agardh C.D. (2001). Classifying diabetes according to the new who clinical stages. Eur. J. Epidemiol..

[37] Sonestedt E., Lyssenko V., Ericson U., Gullberg B., Wirfalt E., Groop L., Orho-Melander M. (2012). Genetic variation in the glucose-dependent insulinotropic polypeptide receptor modifies the association between carbohydrate and fat intake and risk of type 2 diabetes in the malmo diet and cancer cohort. J. Clin. Endocrinol. Metab..

[38] Hanas R., John G. (2010). 2010 consensus statement on the worldwide standardization of the hemoglobin a1c measurement. Pediatr. Diabetes.

[39] Hoelzel W., Weykamp C., Jeppsson J.O., Miedema K., Barr J.R., Goodall I., Hoshino T., John W.G., Kobold U., Little R. (2004). Ifcc reference system for measurement of hemoglobin a1c in human blood and the national standardization schemes in the United States, Japan, and Sweden: A method-comparison study. Clin. Chem..

[40] Rosvall M., Persson M., Ostling G., Nilsson P.M., Melander O., Hedblad B., Engstrom G. (2015). Risk factors for the progression of carotid intima-media thickness over a 16-year follow-up period: The malmo diet and cancer study. Atherosclerosis.

[41] Callmer E., Riboli E., Saracci R., Akesson B., Lindgarde F. (1993). Dietary assessment methods evaluated in the malmo food study. J. Intern. Med..

[42] Elmstahl S., Riboli E., Lindgarde F., Gullberg B., Saracci R. (1996). The malmo food study: The relative validity of a modified diet history method and an extensive food frequency questionnaire for measuring food intake. Eur. J. Clin. Nutr..

[43] Riboli E., Elmstahl S., Saracci R., Gullberg B., Lindgarde F. (1997). The malmo food study: Validity of two dietary assessment methods for measuring nutrient intake. Int. J. Epidemiol..

[44] Wirfalt E., Mattisson I., Johansson U., Gullberg B., Wallstrom P., Berglund G. (2002). A methodological report from the malmo diet and cancer study: Development and evaluation of altered routines in dietary data processing. Nutr. J..

[45] Manjer J., Elmstahl S., Janzon L., Berglund G. (2002). Invitation to a population-based cohort study: Differences between subjects recruited using various strategies. Scand. J. Public Health.

[46] Richardson M.T., Leon A.S., Jacobs D.R., Ainsworth B.E., Serfass R. (1994). Comprehensive evaluation of the minnesota leisure time physical activity questionnaire. J. Clin. Epidemiol..

[47] Mattisson I., Wirfalt E., Aronsson C.A., Wallstrom P., Sonestedt E., Gullberg B., Berglund G. (2005). Misreporting of energy: Prevalence, characteristics of misreporters and influence on observed risk estimates in the malmo diet and cancer cohort. Br. J. Nutr..

[48] Sonestedt E., Wirfalt E., Gullberg B., Berglund G. (2005). Past food habit change is related to obesity, lifestyle and socio-economic factors in the malmo diet and cancer cohort. Public Health Nutr..

[49] Malik V.S., Hu F.B. (2012). Sweeteners and risk of obesity and type 2 diabetes: The role of sugar-sweetened beverages. Curr. Diabetes Rep..

